# BAP1 and PBRM1 Loss Is Associated with Aggressive Clinicopathological Features in Clear Cell Renal Cell Carcinoma: Prognostic Implications in a 10-Year Surgical Cohort

**DOI:** 10.3390/diagnostics16121933

**Published:** 2026-06-22

**Authors:** Mario Daniel Tapia-Tapia, Daniel Sánchez-Zalabardo, Jorge Caño-Velasco, Marcos Torres-Roca, Sara Esparza-Alamanzón, María Rodríguez-Gómez, Eduardo Miraval-Wong, Jaione García-Martínez, Vanesa Ocon-Cruz, Felipe Villacampa-Aubá, Carmina Alejandra Muñoz-Bastidas, Daniel González-Padilla, Julián Sanz-Ortega, Bernardino Miñana-López

**Affiliations:** 1 Department of Urology, Clínica Universidad de Navarra, 31008 Pamplona, Spain; mdtapia@unav.es (M.D.T.-T.); dsanchezz@unav.es (D.S.-Z.); jcanovelasc@unav.es (J.C.-V.); mtroca@unav.es (M.T.-R.); sesparzaa@unav.es (S.E.-A.); mrodriguezgo@unav.es (M.R.-G.); 2 Department of Pathology, Clínica Universidad de Navarra, 31008 Pamplona, Spain; emiraval@unav.es (E.M.-W.); jaionegarcia@unav.es (J.G.-M.); vocon@unav.es (V.O.-C.); 3 Department of Urology, Clínica Universidad de Navarra, 28027 Madrid, Spain; fvauba@unav.es (F.V.-A.); cmunozbasti@unav.es (C.A.M.-B.); dgonzalezp@unav.es (D.G.-P.); 4 Department of Pathology, Clínica Universidad de Navarra, 28027 Madrid, Spain; jsanzo@unav.es

**Keywords:** clear cell renal cell carcinoma, BAP1, PBRM1, immunohistochemistry, biomarkers, prognosis, risk stratification, survival analysis, precision medicine

## Abstract

**Background/Objectives**: Clear cell renal cell carcinoma (ccRCC) is a biologically heterogeneous disease. Beyond VHL inactivation, alterations in chromatin remodeling genes BAP1 and PBRM1 define distinct tumor phenotypes with prognostic implications. We sought to characterize the clinicopathological features and oncological outcomes associated with IHC-defined loss of these markers in a contemporary surgical cohort. **Methods**: We retrospectively analyzed 214 patients undergoing partial or radical nephrectomy for ccRCC (2010–2021). Loss of BAP1 and PBRM1 expression was assessed by automated immunohistochemistry. Tumors with retained expression were classified as wild-type and compared with those showing loss of at least one marker. Survival outcomes were evaluated using Kaplan–Meier analysis, multivariable Cox models, and Restricted Mean Survival Time (RMST). **Results**: IHC-defined loss was identified in 19 patients (8.9%): BAP1 in 12 (5.6%) and PBRM1 in 7 (3.3%). Tumors with IHC-defined loss showed more aggressive features, including larger size (7.7 vs. 4.7 cm; *p* = 0.009), higher necrosis (36.8% vs. 18.5%; *p* = 0.050), and more advanced stage (pT3–pT4: 47.4% vs. 16.4%; *p* < 0.001). Kaplan–Meier analysis demonstrated significantly worse survival outcomes in the IHC-loss group across all endpoints (*p* ≤ 0.011). RMST analysis at 60 months confirmed significantly worse outcomes across all endpoints (*p* ≤ 0.005). **Conclusions**: Loss of BAP1 or PBRM1 identifies a biologically aggressive ccRCC subset with worse oncological outcomes. IHC-based molecular profiling is a practical and accessible tool for risk stratification in surgically treated ccRCC.

## 1. Introduction

Renal cell carcinoma (RCC) remains a significant global health burden, currently ranked as the 9th most frequent neoplasm in men [1]. In 2020 alone, over 431,288 new cases were diagnosed worldwide, with approximately one-third occurring in Europe [2]. Among the various histological variants, clear cell renal cell carcinoma (ccRCC) is the predominant subtype, accounting for 75% to 80% of all diagnoses [3]. This malignancy is characterized by a high degree of clinical and biological heterogeneity; while many cases remain localized and indolent, a substantial proportion of patients experience rapid progression and poor outcomes [4].

At the molecular level, ccRCC is fundamentally linked to alterations in the short arm of chromosome 3 (3p). The most characteristic initiating event is inactivation of the VHL (von Hippel–Lindau) tumor suppressor gene, requiring loss of both alleles [5,6]. While VHL loss is a necessary truncal event, it is often insufficient for full malignant transformation [7,8]. Progression depends on secondary mutations in cooperating driver genes on 3p, most notably PBRM1 (40–50%), SETD2 (10–15%), and BAP1 (10–15%) [5,7]. These genes encode proteins essential for chromatin remodeling and histone modification [5,7]. Their somatic inactivation leads to chromosomal instability and altered gene expression, correlating clinically with higher-grade tumors and reduced overall survival [7,9].

This genotypic diversity determines distinct tumor phenotypes: PBRM1 loss is generally associated with low-grade, angiogenic tumors, whereas BAP1 loss defines a highly aggressive subtype characterized by high-grade features, mTORC1 activation, and an inflamed tumor microenvironment [7,8]. Identification of these alterations may not only detect biologically aggressive tumors in patients clinically classified as low risk, but also predict response to systemic therapies [7,10]. This molecular heterogeneity likely contributes to the significant variability in outcomes among surgically treated patients—approximately 30% of whom recur despite curative-intent resection—and highlights the limitations of traditional clinicopathological factors (TNM stage, grade, necrosis) in fully capturing individual prognosis [11].

Consequently, there is an urgent need to identify molecular biomarkers that better stratify risk and guide therapeutic decisions [9,10]. Systematic IHC assessment of BAP1 and PBRM1 loss offers a promising strategy to identify aggressive tumors in patients otherwise classified as low risk [7,11]. Validation studies confirm that loss of nuclear protein expression reflects loss-of-function mutations in 95% of BAP1-negative and 90% of PBRM1-negative tumors [12,13]. BAP1 IHC shows high diagnostic accuracy, with positive and negative predictive values of 92% and 98%, respectively [7]. Furthermore, automated IHC provides an accessible alternative to whole-exome sequencing, enabling direct risk stratification from formalin-fixed paraffin-embedded specimens [14].

Based on these premises, this study aims to retrospectively analyze the prognostic and predictive implications of BAP1 and PBRM1 protein loss in a large institutional ccRCC cohort, refining risk stratification and supporting personalized renal oncology.

## 2. Materials and Methods

### 2.1. Study Design and Patient Cohort

The present study was designed as a retrospective single-center cohort study conducted at a tertiary academic institution (Clínica Universidad de Navarra, Pamplona, Spain). The primary objective was to investigate the prevalence and prognostic significance of BAP1 and PBRM1 protein loss in patients with clear cell renal cell carcinoma (ccRCC).

The initial study population comprised 220 patients who underwent surgical treatment for renal masses between 2010 and 2021. To ensure a homogeneous and reliable cohort for oncological analysis, the following inclusion criteria were applied: patients undergoing radical or partial nephrectomy, availability of formalin-fixed paraffin-embedded (FFPE) tissue of sufficient quality for histopathological and immunohistochemical analysis, and a minimum clinical follow-up of 5 years to adequately assess long-term survival and recurrence patterns. After applying these criteria, 214 patients with ccRCC were included in the oncological outcomes analysis, following the exclusion of 6 cases due to insufficient tissue or incomplete clinical data. The cohort was characterized by a predominance of early-stage tumors, with most patients presenting with localized disease at diagnosis.

### 2.2. Pathological Assessment and Immunohistochemistry

To maintain diagnostic consistency, all surgical specimens were retrieved from the pathology archive and underwent centralized review by a senior uropathologist following WHO criteria (5th edition, 2022) [15]. Diagnosis of ccRCC was confirmed by identifying characteristic morphological features, including solid, nested, alveolar, or hemorrhagic architectures composed of cells with clear cytoplasm and a delicate branching (“chicken-wire”) vasculature. In cases with atypical or equivocal morphology, validation was performed using carbonic anhydrase IX (CAIX) immunohistochemistry, requiring diffuse and intense membranous positivity.

After centralized pathological review, 198 cases were definitively confirmed as ccRCC and had tissue of sufficient quality for new IHC staining of BAP1 and PBRM1. For the remaining 16 patients, IHC results were retrieved from original diagnostic reports performed using identical antibody clones and the same automated platform, re-reviewed by the study uropathologist (E.M.W.) to ensure consistency. All 214 patients were included in the analyses.

From each selected block, a serial 3 μm unstained paraffin-embedded section was obtained and submitted for IHC staining using a validated and previously published method [16]. All stains were performed on the Roche BENCHMARK ULTRA automated platform (Roche Diagnostics, Basel, Switzerland). BAP1 immunostaining was performed using a mouse monoclonal anti-BAP1 antibody (clone C-4; Santa Cruz Biotechnology, Inc., Santa Cruz, CA, USA; catalog no. sc-28383) at a dilution of 1:50, incubated for 48 min at 37 °C. Antigen retrieval was performed using CC1 high pH buffer at 95 °C for 92 min. Signal detection was carried out using the ultraView detection system (Roche Diagnostics) with amplification (Amplification Kit, catalog no. 05266114001, Roche Diagnostics), according to the manufacturer’s protocol. PBRM1 immunostaining was performed using a rabbit polyclonal anti-PBRM1 antibody (Bethyl Laboratories, Inc., Montgomery, TX, USA; catalog no. A301-591A) at a dilution of 1:250, incubated for 32 min at 37 °C. Antigen retrieval was performed using CC1 high pH buffer at 95 °C for 36 min. Signal detection was performed using the ultraView detection system without amplification.

**Figure 1:**
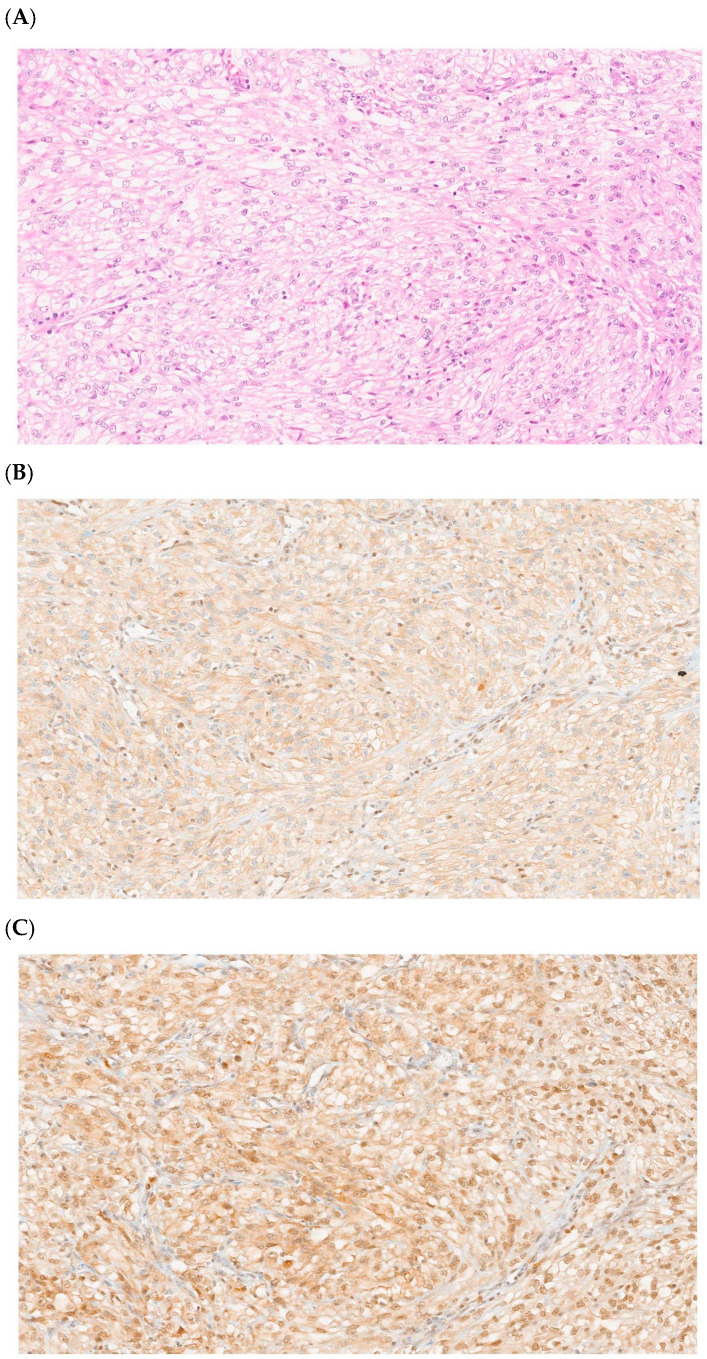
Representative immunohistochemical staining (clear cell renal cell carcinoma, 20× magnification). (**A**) Hematoxylin and eosin (H&E) staining showing clear cell morphology. (**B**) BAP1 loss: complete absence of nuclear staining in tumor cells with retained expression in stromal cells and lymphocytes (internal positive controls). (**C**) PBRM1 preserved expression: diffuse and strong nuclear staining in tumor cells.

**Figure 2:**
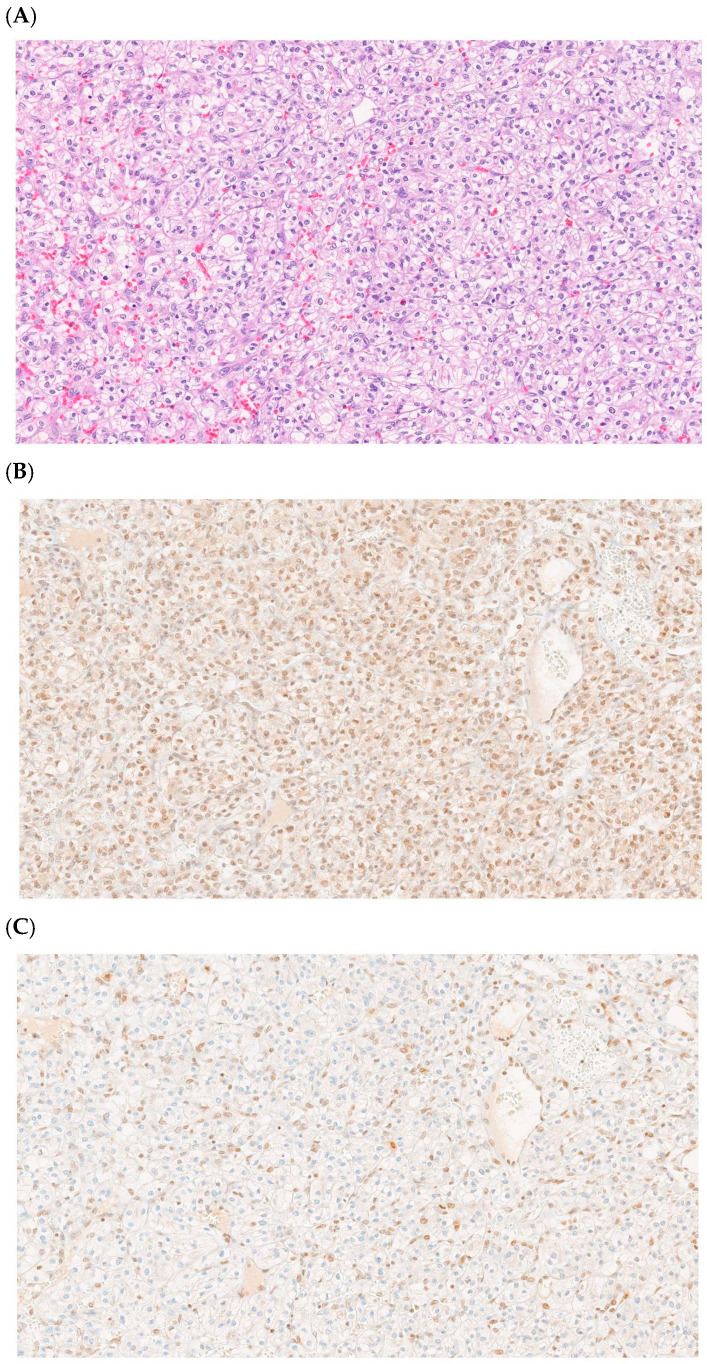
Representative immunohistochemical staining (clear cell renal cell carcinoma, 20× magnification). (**A**) Hematoxylin and eosin (H&E) staining showing clear cell morphology. (**B**) BAP1 preserved expression: diffuse and strong nuclear staining in tumor cells. (**C**) PBRM1 loss: complete absence of nuclear staining in tumor cells with retained expression in stromal cells and lymphocytes (internal positive controls).

Each slide was independently reviewed by a dedicated uropathologist to assess BAP1 and PBRM1 expression status. Positivity was defined as strong and diffuse nuclear staining in tumor cells. Loss of expression was defined as complete absence of nuclear staining in tumor cells; cases with weak, focal, or heterogeneous staining were carefully reviewed and classified as IHC-defined loss only when diffuse absence was confirmed. Internal positive controls required retained nuclear staining in stromal cells, endothelial cells, and intratumoral lymphocytes; slides lacking this background staining were excluded. External positive and negative controls were selected according to The Human Protein Atlas (www.proteinatlas.org) [17]. Representative immunohistochemical findings showing BAP1 loss and preserved PBRM1 expression are shown in Figure 1. Representative cases with preserved BAP1 expression and PBRM1 loss are presented in Figure 2.

### 2.3. Clinical Data Collection and Statistical Analysis

A comprehensive institutional database was constructed integrating clinical and histopathological variables. Baseline characteristics included age at surgery, sex, smoking status, and pre-operative hemoglobin levels. Pathological variables recorded during centralized review included maximum tumor size (cm), microscopic coagulative tumor necrosis, and vascular involvement. Tumors were staged according to the AJCC TNM classification (8th edition, 2017), and nuclear grade was assigned using the WHO/ISUP grading system (2012), dichotomized into low (1–2) and high grade (3–4). Postoperative renal function was assessed using creatinine clearance. The cohort was stratified according to molecular profile into wild-type tumors and tumors with IHC-defined loss of BAP1 and/or PBRM1 expression (hereafter referred to as “IHC-loss” group for brevity). It is important to note that throughout this manuscript the terms “IHC-loss” or “mutation” refer to IHC-defined protein loss, a validated surrogate for loss-of-function mutations [12,13], rather than direct genotyping. BAP1 and PBRM1 were analyzed as a combined group rather than separately due to the limited number of individual cases with IHC-defined loss (BAP1: *n* = 12; PBRM1: *n* = 7), which precluded statistically reliable separate analyses. We acknowledge that these markers have distinct biological phenotypes—BAP1 loss is associated with an inflamed/proliferative phenotype, whereas PBRM1 loss defines a predominantly angiogenic phenotype—and that pooling represents a pragmatic compromise driven by sample size constraints, as discussed in the Limitations section.

Qualitative variables were compared using the chi-squared test, while quantitative variables were compared using Student’s *t*-test. Oncological outcomes included progression-free survival (PFS), defined as time from surgery to recurrence or metastasis; cancer-specific survival (CSS), defined as time from surgery to death attributable to ccRCC; and overall survival (OS), defined as time from surgery to death from any cause. Patients without events were censored at last follow-up. Survival probabilities were estimated using the Kaplan–Meier method and compared using the log-rank test. Logistic and Cox proportional hazards models were used to evaluate IHC expression status as an independent predictor, adjusting for tumor stage, size, grade, and necrosis. A *p*-value < 0.050 was considered statistically significant. Statistical analyses were performed using IBM SPSS Statistics version 30.0. Based on published data indicating 16–24% survival differences associated with BAP1/PBRM1 loss [7,14], a theoretical sample size estimation suggests that approximately 150 patients would be required to achieve 80% power (α = 0.050) to detect a hazard ratio of 2.0 for cancer-specific mortality. Although no formal a priori calculation was performed due to the retrospective design, the final cohort size was considered comparable to previously published institutional biomarker studies in ccRCC. As a sensitivity analysis to address the instability of Kaplan–Meier median estimates, Restricted Mean Survival Time (RMST) was calculated at 60 months for progression-free survival (PFS), cancer-specific survival (CSS), and overall survival (OS). RMST was estimated as the area under the Kaplan–Meier curve up to the truncation point, and the significance of the RMST difference between groups was assessed using a permutation test (1000 iterations). Results are presented in Supplementary Figure S1.

## 3. Results

### 3.1. Study Cohort and Baseline Characteristics

The study population comprised 214 patients with ccRCC treated with partial or radical nephrectomy. The cohort had a mean age of 58.9 years (range 26–89) and a mean BMI of 27.2; 44.9% were active smokers and 36.4% former smokers. Mean hemoglobin decreased from 13.94 g/dL pre-operatively to 11.58 g/dL post-operatively. Mean serum creatinine increased from 1.04 to 1.25 mg/dL, with a decline in creatinine clearance from 82.19 to 65.69 mL/min.

Tumors were predominantly early stage, with clinical T stage distribution of cT1 (69.6%), cT2 (10.3%), cT3 (18.7%), and cT4 (1.4%). Most patients had localized disease (cN0 99.1%, cM0 94.9%), with nodal involvement in 0.9% and metastases in 5.1%. Mean tumor size was 4.92 cm, with pathological stage distribution of pT1 (72.8%), pT2 (8.0%), pT3 (17.8%), and pT4 (1.4%). Nuclear grade II predominated (59.8%), with tumor necrosis present in 20.1% and microvascular invasion in 9.3%.

During follow-up, 47 patients (21.9%) developed recurrence or progression. Of these, 9 (19.1%) underwent local salvage procedures, including adrenalectomy (*n* = 3), partial nephrectomy (*n* = 4), and radiofrequency ablation (*n* = 2), while 38 (82.6%) required systemic therapy, of whom 34 completed treatment. Tyrosine kinase inhibitors were the most frequently used agents (50%, *n* = 19), including sunitinib (26.3%), pazopanib (21.1%), and cabozantinib (2.6%). Immune checkpoint inhibitors were administered in 36.8% (nivolumab 18.4%, atezolizumab 10.5%, pembrolizumab 7.9%), while everolimus was used in one case (2.6%) and radiotherapy in 10.5% (*n* = 4).

### 3.2. Molecular Profile and Prevalence of IHC-Loss

Out of the 214 patients analyzed, 195 (91.1%) were identified as wild-type, showing retained nuclear expression of both proteins. Conversely, 19 patients (8.9%) demonstrated loss of expression of at least one marker. Within this IHC-loss subgroup, 12 cases (5.6%) exhibited BAP1 loss, while 7 cases (3.3%) exhibited PBRM1 loss.

### 3.3. Baseline Clinical Characteristics and Comparative Analysis

Baseline clinical comparisons revealed significant demographic differences between groups. Patients harboring IHC-loss were significantly older at the time of surgery, with a mean age of 64.4 years compared to 58.4 years in the wild-type group (*p* = 0.010). Furthermore, IHC-loss cases had lower preoperative hemoglobin levels (12.9 g/dL vs. 14.0 g/dL; *p* = 0.005).

The study groups were otherwise comparable with respect to baseline clinical and surgical variables, with no significant differences in smoking habits, lymph node involvement (pN), or surgical margin status (*p* > 0.050). Regarding the observation period, the mean follow-up time was 51.1 months in the IHC-loss group, compared with 75.1 months in the wild-type group (*p* = 0.034). Baseline characteristics are summarized in Table 1.

### 3.4. Histopathological Characteristics of IHC-Loss ccRCC

The presence of BAP1 or PBRM1 alterations was associated with an aggressive morphological phenotype. IHC-loss tumors exhibited a significantly larger mean tumor size (7.7 cm maximum diameter) than tumors with retained expression (4.7 cm; *p* = 0.009). Several histopathological parameters indicated a higher degree of biological aggression in the IHC-loss group:

- Tumor Necrosis: Microscopic coagulative necrosis was detected in 7 of 19 IHC-loss tumors (36.8%), whereas it was present in 36 of 195 wild-type tumors (18.5%) (*p* = 0.050; borderline significance).
- Vascular Involvement: The frequency of vascular invasion was markedly higher in tumors with protein loss, occurring in 5 of 19 IHC-loss cases (26.3%) compared with 15 of 195 wild-type tumors (7.7%) (*p* = 0.008).
- Staging: IHC-loss was strongly associated with advanced pathological stages. Nearly half of the IHC-loss group (9 of 19 patients; 47.4%) presented with pT3–pT4 disease, compared to 32 of 195 patients (16.4%) in the wild-type group (*p* < 0.001).
- Nuclear Grade: A strong correlation was observed with high-grade pathology. 8 of 19 tumors (42.1%) with BAP1 or PBRM1 loss were classified as nuclear Grade 3–4, compared to 25 of 195 tumors (12.8%) in the wild-type group (*p* < 0.001). Histopathological characteristics are summarized in Table 2.

### 3.5. Impact on Renal Function and Surgical Outcomes

Post-surgical renal function, measured by creatinine clearance, was significantly impaired in the IHC-loss group. The mean clearance was 57.1 mL/min in patients with IHC-loss versus 66.5 mL/min in those without (*p* = 0.030). No significant differences were observed between the two groups for smoking habits, lymph node involvement (pN1), or positive surgical margins.

### 3.6. Long-Term Oncological Progression

The clinical evolution following surgery showed a higher risk of disease progression among patients with loss of BAP1 and/or PBRM1 expression. Disease progression occurred in 9 of 19 patients (47.4%) with protein loss compared with 38 of 195 patients (19.5%) in the wild-type group (*p* = 0.004). Among patients who developed recurrence, those in the IHC-loss group had a higher rate of progression after salvage treatment than those in the wild-type group (8 of 9 patients [88.9%] vs. 26 of 38 [68.4%]); however, this difference did not reach statistical significance (*p* = 0.496).

### 3.7. Survival Analysis

**Figure 3:**
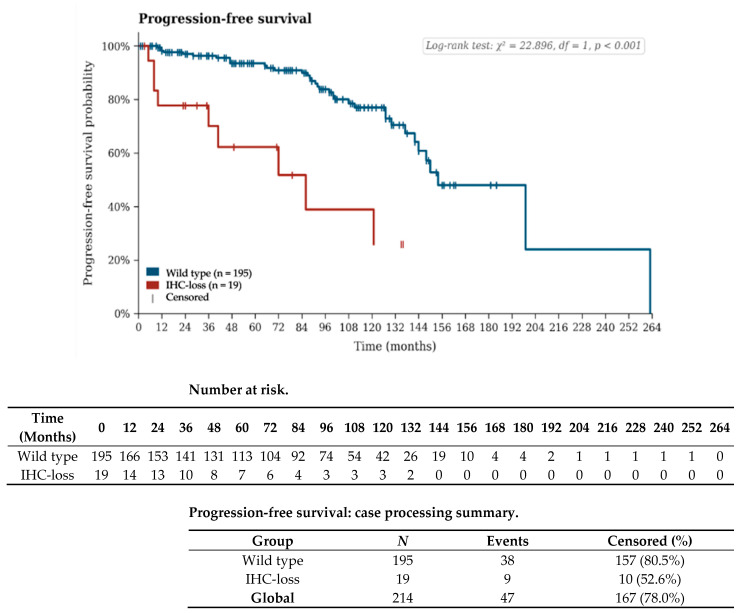
Kaplan–Meier curves for progression-free survival. Tick marks indicate censored observations. Log-rank (Mantel–Cox) test: χ^2^ = 22.896, df = 1, *p* < 0.001. The number at risk table shows patients remaining under observation at each 12-month interval.

**Figure 4:**
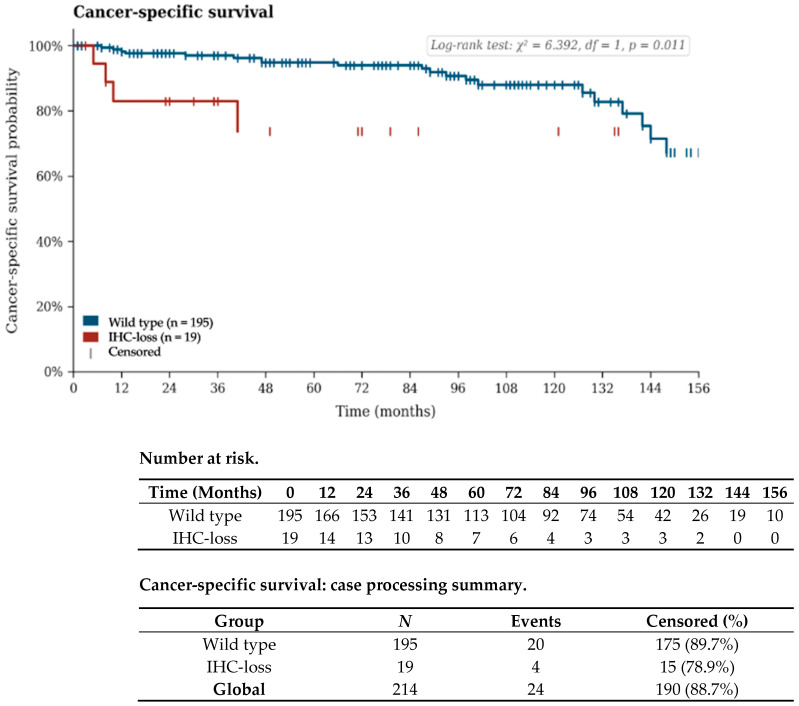
Kaplan–Meier curves for cancer-specific survival. Tick marks indicate censored observations. Log-rank (Mantel–Cox) test: χ^2^ = 6.392, df = 1, *p* = 0.011. The number at risk table shows patients remaining under observation at each 12-month interval.

**Figure 5:**
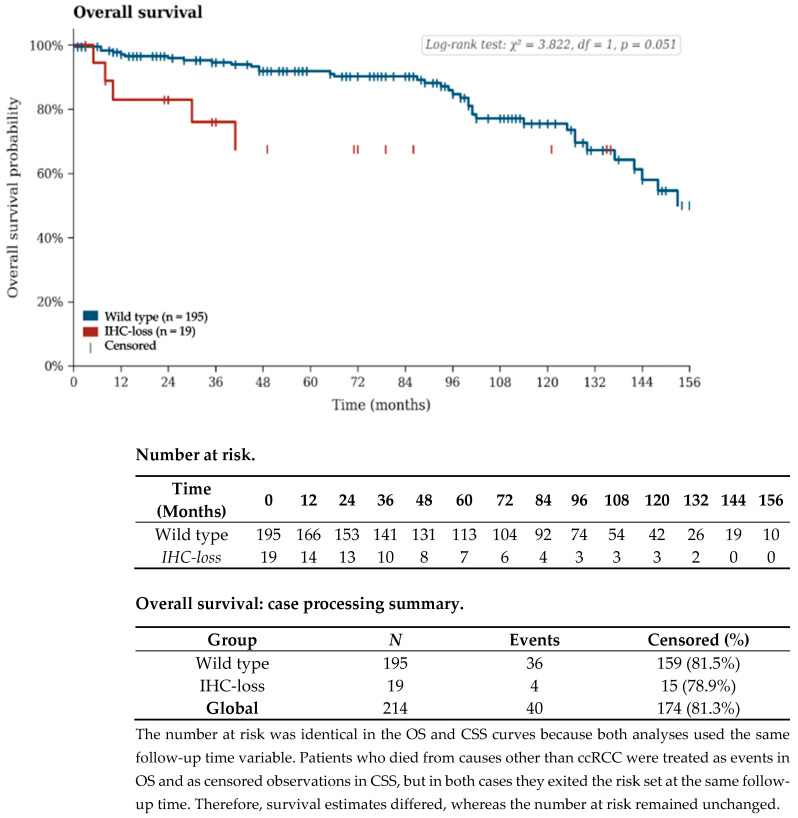
Kaplan–Meier curves for overall survival. Tick marks indicate censored observations. Log-rank (Mantel–Cox) test: χ^2^ = 3.822, df = 1, *p* = 0.051. The number at risk table shows patients remaining under observation at each 12-month interval.

The presence of BAP1 or PBRM1 IHC-defined loss was associated with worse survival trends across all endpoints, reaching statistical significance for PFS and CSS. The mean PFS was 69.5 months in the IHC-loss group vs. 143.5 months in the wild-type group (*p* < 0.001); mean CSS was 105.4 vs. 213.6 months (*p* = 0.011); and mean OS was 98.8 vs. 183.5 months (*p* = 0.051). The median PFS was 46.0 months in the IHC-loss group, while the wild-type group had not reached the median at the end of follow-up. Median CSS was not reached in either group. Median OS was not reached in the IHC-loss group and was 153.0 months in the wild-type group. Kaplan–Meier survival curves are shown in Figure 3, Figure 4 and Figure 5. As a robustness check avoiding extrapolation beyond the observation period, RMST at 60 months was: PFS 35.4 vs. 52.8 months (difference 17.4 months, *p* = 0.001); CSS 49.3 vs. 58.3 months (difference 9.0 months, *p* = 0.002); OS 47.4 vs. 57.2 months (difference 9.8 months, *p* = 0.005). Full RMST curves are presented in Supplementary Figure S1.

### 3.8. Prognostic Factors for Disease Progression

In the initial univariable analysis of the entire cohort (*n* = 214), the presence of BAP1 or PBRM1 IHC-loss was associated with a 3.8-fold increased risk of disease progression following surgery (OR = 3.8; *p* = 0.006). However, after multivariable adjustment for tumor size, nuclear grade, and clinical TNM stage, this molecular effect lost statistical significance (*p* > 0.050). In the multivariable model, TNM stage was the only independent predictor of progression (*p* = 0.041). Prognostic factors for disease progression are presented in Table 3.

A stratified analysis of locally advanced disease (pT3–pT4, *n* = 40) was also performed. Given the small number of events (4 CSS deaths in the IHC-loss arm), this analysis is presented strictly as exploratory in Supplementary Tables S2 and S3. Briefly, patients with IHC-defined marker loss in this subgroup had a median CSS of 41.0 months compared to 137.0 months in wild-type patients. In a reduced multivariable Cox model, IHC-defined marker loss showed a suggestive association with cancer-specific mortality (HR = 6.17, 95% CI 1.3–29.2, *p* = 0.022); however, these results must be interpreted strictly as hypothesis-generating given the events-per-variable limitation (≥10 events required; only 4 available).

## 4. Discussion

The observed IHC-loss prevalence (5.6% for BAP1 and 3.3% for PBRM1) was lower than that reported in large international series (10–15% and 40–50%, respectively) [5,18]. However, this discrepancy can be explained by the specific pathological composition of our cohort and by the evolutionary dynamics of these genetic alterations. While many high-impact studies analyze cohorts enriched with high-risk or metastatic patients, the present study represents a consecutive surgical series in which most cases (72.8%) were diagnosed at an early pT1 stage. The literature focused specifically on metastatic ccRCC reports substantially higher rates of protein loss, including 20% for BAP1 and 57% for PBRM1 in primary tumors from matched metastatic pairs [14], and even up to 62.1% BAP1 loss when metastatic tissue is analyzed directly [19].

Moreover, the distinction between truncal and branched (subclonal) mutations provides a critical biological framework to interpret these variations. According to evolutionary models such as TRACERx, while VHL inactivation represents a near-universal truncal event [5], BAP1 and PBRM1 alterations may occur either as early truncal drivers or as later branched mutations [14]. Subclonal events are considerably more prevalent in advanced, heterogeneous, or metastatic tumors [14,20]. In our study, automated immunohistochemistry requiring diffuse nuclear loss likely prioritizes truncal alterations, potentially excluding branched mutations presenting as focal negative areas, which are more common in advanced cohorts [12,14]. Therefore, the prevalence observed reflects a population predominantly composed of early-stage tumors in which these aggressive epigenetic drivers may not yet have become ubiquitous through clonal expansion.

The molecular classification of ccRCC based on IHC loss of BAP1 and PBRM1 identifies a distinct high-risk subset characterized by aggressive biological behavior [14]. Our institutional findings confirm that these epigenetic alterations are associated with significantly larger mean tumor size (7.7 vs. 4.7 cm), higher frequency of high nuclear grades (42.1% G3–G4 vs. 12.8%), and greater prevalence of tumor necrosis (36.8% vs. 18.5%) and microvascular involvement (26.3% vs. 7.7%).

These results are consistent with established evidence indicating that loss of these chromatin remodelers acts as a truncal driver of an aggressive phenotype early in tumor evolution [8,14,20]. Specifically, BAP1 loss has been associated with rhabdoid differentiation, high-grade nuclei, and an inflamed tumor microenvironment, whereas PBRM1 loss is more frequently linked to classic low-grade clear cell morphology but carries a higher risk of late-stage progression [8,14,21]. Multiple studies have demonstrated that absence of BAP1 or PBRM1 expression correlates with adverse oncological outcomes, including significantly reduced cancer-specific survival and increased risk of disease recurrence [7,12,14].

Furthermore, IHC-loss cases had significantly lower preoperative hemoglobin levels (12.9 vs. 14.0 g/dL), a systemic marker commonly associated with increased inflammatory burden and poor prognosis in aggressive renal malignancies [8,19]. No significant differences were observed regarding smoking status, lymph node involvement, or positive surgical margins, suggesting that disparities in clinical outcomes are primarily driven by intrinsic tumor biology.

The analysis of oncological outcomes underscores the substantial adverse prognostic impact associated with BAP1 and PBRM1 loss. In the overall population, patients harboring these alterations demonstrated significantly inferior outcomes across all survival metrics. However, this association was no longer statistically significant in the multivariable model after adjustment for established prognostic variables, suggesting that the prognostic effect of IHC-defined loss may be largely mediated through the aggressive clinicopathological phenotype it promotes—namely higher stage and grade—rather than acting as an independent determinant of survival, consistent with the current biological understanding of these genes as truncal drivers [4,14].

The most clinically relevant finding of this exploratory analysis emerged from the stratified analysis of locally advanced disease (pT3–pT4, *n* = 40). Within this high-risk subgroup, patients with IHC-defined marker loss faced a dramatically accelerated clinical course, with a median CSS of only 41.0 months compared to 137.0 months for wild-type patients. In the reduced multivariable Cox regression model for this subgroup, IHC-defined marker loss showed a suggestive association with cancer-specific mortality (HR = 6.17, 95% CI 1.3–29.2, *p* = 0.022). However, this finding must be interpreted strictly as exploratory and hypothesis-generating: the model was based on only 4 CSS events in the IHC-loss arm, which falls well below the minimum events-per-variable threshold required for reliable Cox regression (≥10 events per variable). The resulting wide confidence interval reflects this statistical instability, and no causal or confirmatory conclusions can be drawn. A parallel multivariable analysis for overall survival in this subgroup did not yield statistical significance, further underscoring the preliminary nature of these findings. Validation in larger, prospective, multi-institutional cohorts is essential before these results can inform clinical practice.

Notably, the significantly shorter mean follow-up in the IHC-loss group (51.1 vs. 75.1 months, *p* = 0.034) deserves careful consideration. While this differential likely reflects the inherently more aggressive clinical behavior of these tumors, it also introduces a potential bias in Kaplan–Meier estimates, as earlier truncation of observation may overestimate differences in survival estimates. It should also be noted that the median PFS in the wild-type group and the median CSS and OS in both groups were not reached at the end of follow-up, reflecting favorable outcomes in most patients. To provide a more robust quantification, RMST at 60 months was: PFS 35.4 vs. 52.8 months (difference 17.4 months, *p* = 0.001); CSS 49.3 vs. 58.3 months (difference 9.0 months, *p* = 0.002); OS 47.4 vs. 57.2 months (difference 9.8 months, *p* = 0.005). These results confirm that survival differences are robust and not merely artifacts of extrapolation (Supplementary Figure S1). Additionally, formal sensitivity analyses such as inverse probability of censoring weighting were not performed, but RMST truncation at 60 months mitigates the bias from unequal follow-up between groups.

The incorporation of BAP1 and PBRM1 IHC into routine pathology reports may represent a practical strategy to improve risk stratification, allowing identification of biologically aggressive phenotypes directly from archival FFPE specimens [14]. In the metastatic or recurrent setting, molecular profiling based on BAP1 and PBRM1 status may help guide treatment selection: BAP1-deficient tumors exhibit an inflamed/proliferative phenotype with higher PD-L1 expression and may derive greater benefit from immune checkpoint inhibitors [14]; the known association between BAP1 inactivation and mTORC1 activation also suggests that mTOR inhibitors may represent a potential therapeutic option, and emerging combinations including CDK4/6 inhibitors paired with immunotherapy are being explored for these highly aggressive subsets [22]. Conversely, PBRM1-deficient tumors display an angiogenic gene signature associated with improved responses to VEGF-targeted therapies; novel agents targeting the hypoxia response, such as HIF-2α inhibitors, are particularly promising given that PBRM1 loss may amplify hypoxia-driven signaling pathways [22]. However, given that these two alterations were analyzed as a combined group in the present study due to sample size constraints, therapeutic recommendations based on pooled results should be interpreted with caution—their distinct and potentially opposing biological implications underscore the need for separate analyses in future larger cohorts. IHC expression does not always fully reflect the underlying molecular alterations, and results from these surrogate markers should therefore be interpreted accordingly [14].

### Limitations

This study has several limitations. First, its retrospective single-center design and the relatively small number of IHC-loss cases may have reduced the statistical power of multivariable analyses. In particular, the subgroup analysis of locally advanced tumors (pT3–pT4) was constrained by sample size, resulting in wide confidence intervals; these findings have been moved to Supplementary Tables S2 and S3 and should be interpreted as strictly exploratory. In addition, BAP1 and PBRM1 alterations were analyzed as a combined group due to the limited number of individual cases (BAP1: *n* = 12; PBRM1: *n* = 7), which precluded reliable separate analyses. We acknowledge that BAP1 and PBRM1 have distinct biological roles, phenotypes, and therapeutic implications—BAP1 loss is associated with an inflamed/proliferative phenotype with potential ICI benefit, whereas PBRM1 loss defines a predominantly angiogenic phenotype with preferential sensitivity to VEGF-targeted therapies [7,14,22]. Pooling these markers is therefore a pragmatic compromise driven by sample size constraints. Descriptive data for each marker separately are provided in Supplementary Table S1 for transparency. Separate analyses in future larger cohorts will be essential to clarify their individual prognostic contributions.

Second, the long study period (2010–2021) spans significant changes in the systemic treatment landscape for renal cell carcinoma, including the introduction of targeted therapies and immune checkpoint inhibitors, which may have influenced survival outcomes.

Furthermore, preoperative hemoglobin levels, which were significantly lower in the IHC-loss group (12.9 vs. 14.0 g/dL; *p* = 0.005) and have been established as an independent prognostic indicator in ccRCC, were not included in the multivariable Cox models. This decision was made to avoid overfitting given the limited number of events, particularly in the pT3–pT4 subgroup (4 CSS deaths), where the reduced model was already constrained to a single variable. The potential confounding effect of preoperative anemia should be addressed in future studies with larger sample sizes. A formal competing-risks analysis was not performed; the Kaplan–Meier CSS estimates may overestimate the cumulative incidence of cancer-specific death in the older IHC-loss group if non-ccRCC mortality is higher.

Despite these limitations, several methodological strengths support the robustness of our findings. All cases underwent centralized pathological review, ensuring diagnostic consistency. In addition, immunohistochemical analyses were performed using standardized protocols with strict quality control, and follow-up data were comprehensive with minimal loss to follow-up.

## 5. Conclusions

Loss of BAP1 or PBRM1 expression, assessed by standardized immunohistochemistry, identifies a biologically aggressive ccRCC subset characterized by larger tumor size, higher nuclear grade, greater necrosis, microvascular involvement, and significantly worse progression-free and cancer-specific survival. RMST analysis confirmed a consistent survival disadvantage across all endpoints within the first 60 months. The accessibility of IHC-based molecular profiling enables integration into routine pathology workflows without the need for genomic sequencing, supporting its potential role in refined risk stratification of surgically treated ccRCC.

All findings from the locally advanced (pT3–pT4) subgroup analysis are presented in Supplementary Tables S2 and S3 and should be considered strictly exploratory and hypothesis-generating. Prospective validation in larger, multi-institutional cohorts is required to confirm the prognostic and predictive value of BAP1/PBRM1 status in ccRCC.

## Figures and Tables

**Table 1:** Baseline characteristics of the study cohort according to IHC expression status.

Variable	Wild Type (*n* = 195)	IHC-Loss (*n* = 19)	*p* Value
Age (years), mean ± SD	58.4 ± 11.5	64.4 ± 12.5	0.010
BMI (kg/m^2^), mean ± SD	27.3 ± 3.9	26.7 ± 4.6	0.520
Current or former smoker, *n* (%)	71 (44.1)	7 (52.6)	0.590
Preoperative hemoglobin (g/dL), mean ± SD	14.0 ± 1.8	12.9 ± 1.8	0.005
Follow-up time (months), mean ± SD	75.1 ± 51.5	51.1 ± 43.6	0.034

SD = standard deviation; BMI = body mass index. Continuous variables are presented as mean ± SD, and categorical variables as number (percentage).

**Table 2:** Histopathological characteristics according to IHC expression status.

Variable	Wild Type (*n* = 195)	IHC-Loss (*n* = 19)	*p* Value
Tumor size (cm), mean ± SD	4.7 ± 3.0	7.7 ± 5.0	0.009
Pathologic T stage (pT3–pT4), *n* (%)	32 (16.4)	9 (47.4)	<0.001
Lymph node involvement (pN+), *n* (%)	4 (2.1)	1 (5.3)	0.376
Nuclear grade (1–2 vs. 3–4), *n* (%)	25 (12.8)	8 (42.1)	<0.001
Tumor necrosis, *n* (%)	36 (18.5)	7 (36.8)	0.050
Microvascular involvement, *n* (%)	15 (7.7)	5 (26.3)	0.008
Surgical margin status (positive), *n* (%)	3 (1.5)	1 (5.3)	0.496

SD = standard deviation. Continuous variables are presented as mean ± SD, and categorical variables as number (percentage).

**Table 3:** Univariable and multivariable logistic regression analyses of prognostic factors for disease progression.

Variable		Univariable OR (95% CI)	*p* Value	Multivariable OR (95% CI)	*p* Value
IHC expression status (Ref. wild type)		3.84 (1.45–10.12) ^1^	0.006	—	—
Nuclear grade (1–2 vs. 3–4)		3.43 (1.55–7.55)	0.002	—	—
Tumor necrosis (Ref. absence of necrosis)		3.67 (1.77–7.61)	<0.001	—	—
Tumor size (cm)		1.28 (1.15–1.41)	<0.001	—	—
Clinical T stage (Ref. T1)	T2	8.03 (2.9–21.9)	<0.001	4.5 (1.2–16.1)	0.021
	T3	9.64 (4.2–22.1)	<0.001	6.5 (2.2–19.2)	<0.001
	T4	19.28 (1.6–226.3)	0.019	—	—
Preoperative hemoglobin (g/dL)		0.79 (0.66–0.94) ^2^	0.009	—	—

Ref = reference category; OR = odds ratio; CI = confidence interval. ^1^ IHC expression status was significant in univariable analysis but did not retain statistical significance after multivariable adjustment. ^2^ OR < 1 indicates a decreased risk per unit increase in the variable; therefore, higher preoperative hemoglobin levels are associated with a lower risk of disease progression. Variables that did not retain statistical significance after multivariable adjustment are indicated by “—”.

## Data Availability

The original contributions presented in this study are included in the article/Supplementary Materials. Further inquiries can be directed to the corresponding author.

## Supplementary Materials

The following supporting information can be downloaded at: https://www.mdpi.com/article/10.3390/diagnostics16121933/s1, Figure S1. Restricted Mean Survival Time (RMST) Analysis; Table S1. Clinicopathological Features According to Individual IHC Marker Loss Status; Table S2. Exploratory Subgroup Analysis—Locally Advanced Disease (pT3–pT4); Table S3. Survival Outcomes and Multivariable Cox Regression Analysis in Patients with Advanced Disease (pT3–pT4).

## Abbreviations

The following abbreviations are used in this manuscript:

AJCC	American Joint Committee on Cancer
BAP1	BRCA1 Associated Protein 1
CAIX	Carbonic Anhydrase IX
ccRCC	Clear Cell Renal Cell Carcinoma
CI	Confidence Interval
CSS	Cancer-Specific Survival
DNA	Deoxyribonucleic Acid
FFPE	Formalin-Fixed Paraffin-Embedded
HIF-2α	Hypoxia-Inducible Factor 2 Alpha
HR	Hazard Ratio
ICI	Immune Checkpoint Inhibitor
IHC	Immunohistochemistry
mTORC1	Mechanistic Target of Rapamycin Complex 1
OR	Odds Ratio
OS	Overall Survival
PD-L1	Programmed Death-Ligand 1
PFS	Progression-Free Survival
RCC	Renal Cell Carcinoma
SPSS	Statistical Package for the Social Sciences
TKIs	Tyrosine Kinase Inhibitors
TNM	Tumor, Node, Metastasis (Staging System)
VEGF	Vascular Endothelial Growth Factor
VHL	von Hippel–Lindau
WHO	World Health Organization
