# Supplementary material for: BAP1 and PBRM1 Loss Is Associated with Aggressive Clinicopathological Features in Clear Cell Renal Cell Carcinoma: Prognostic Implications in a 10-Year Surgical Cohort
Source: Diagnostics (Basel). 2026 Jun 22;16(12):1933. doi: 10.3390/diagnostics16121933 (PMC13298600; doi:10.3390/diagnostics16121933)
Supplement: Supplementary file 1 [file diagnostics-16-01933-s001.zip › diagnostics-4263267-supplementary.pdf]

diagnostics

Article

BAP1 and PBRM1 Loss Is Associated with Aggressive Clinicopathological Features in Clear Cell Renal Cell Carcinoma: Prognostic Implications in a 10-Year Surgical Cohort.

Mario Daniel Tapia-Tapia, Daniel Sánchez-Zalabardo, Bernardino Miñana-López et al.

Supplementary Material

Figure S1. Restricted Mean Survival Time (RMST) Analysis

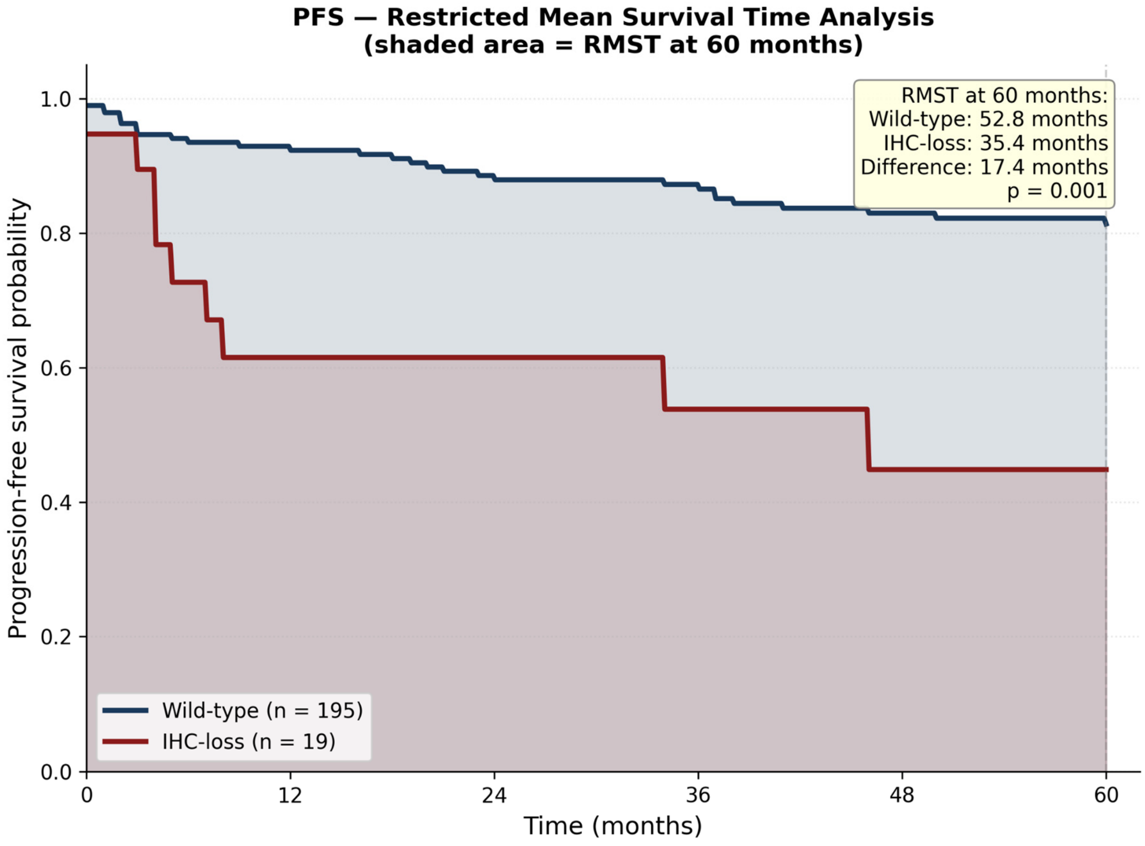

p-value from permutation test (1,000 iterations) for RMST difference. Difference = Wild-type RMST − IHC-loss RMST.

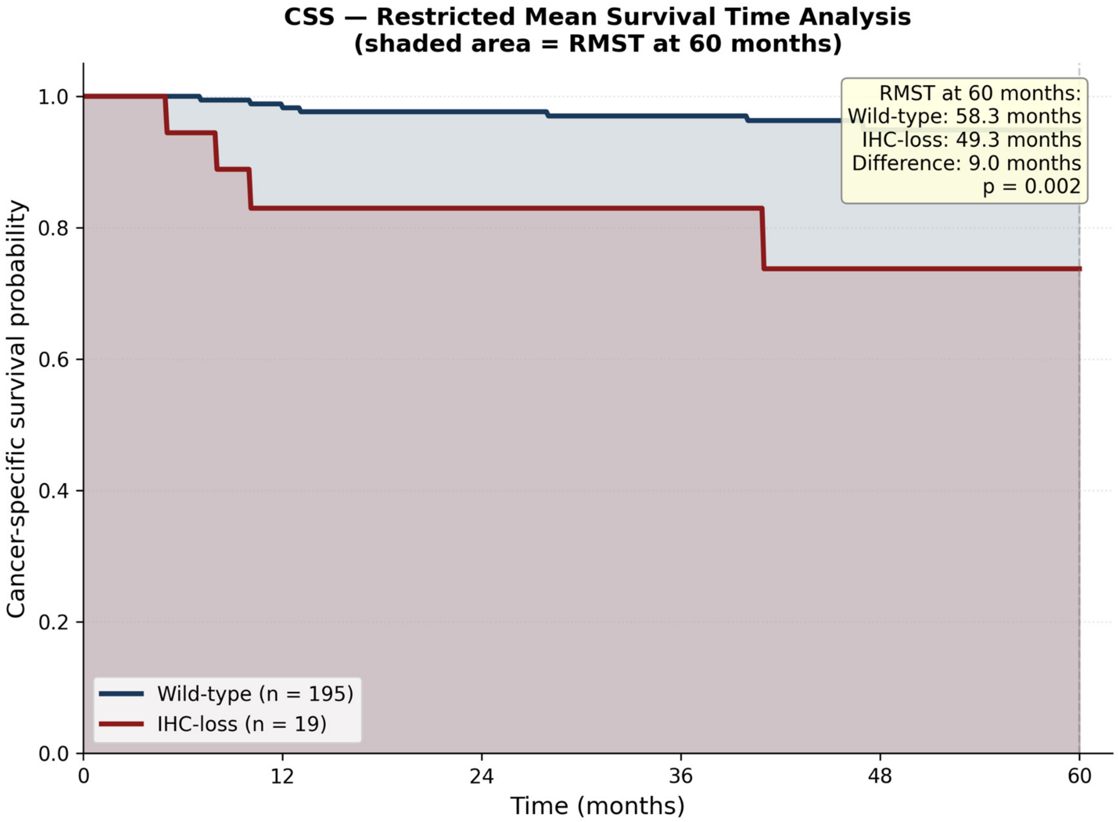

p-value from permutation test (1,000 iterations) for RMST difference. Difference = Wild-type RMST − IHC-loss RMST.

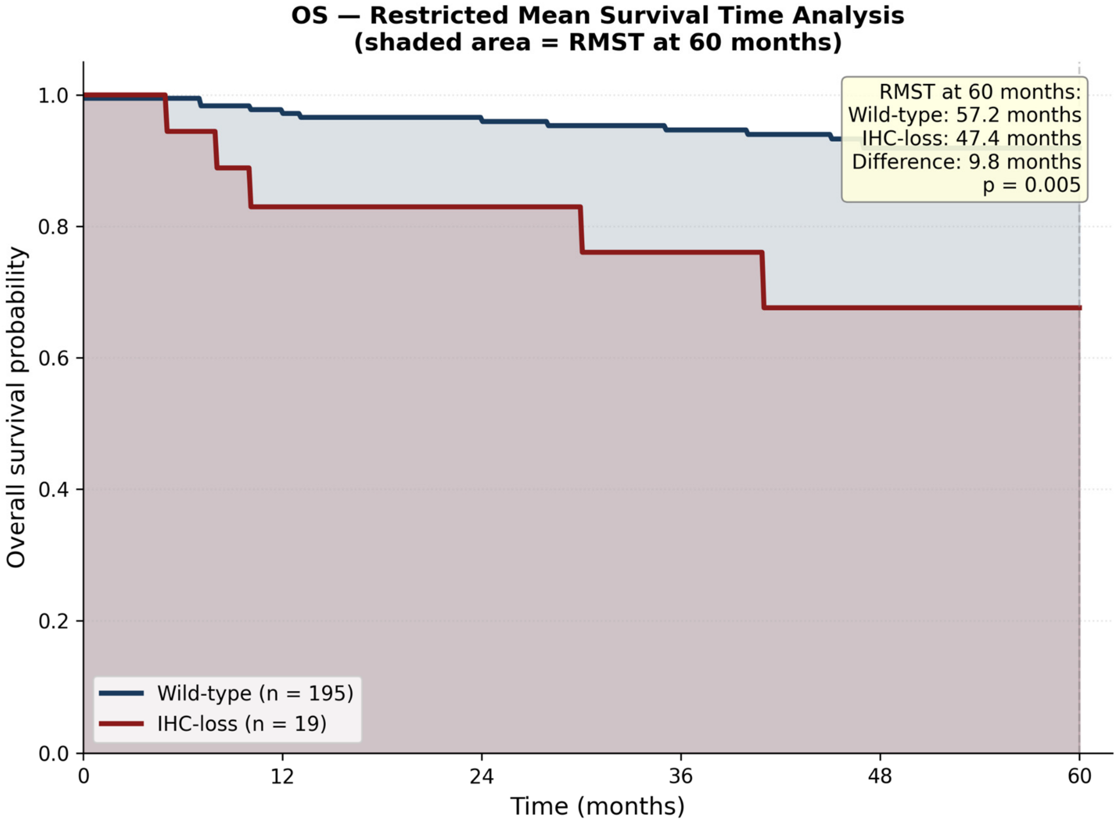

p-value from permutation test (1,000 iterations) for RMST difference. Difference = Wild-type RMST − IHC-loss RMST.

Figure S1. Restricted Mean Survival Time (RMST) analysis for progression-free survival (PFS), cancer-specific survival (CSS), and overall survival (OS) at 60 months. The shaded area under each Kaplan–Meier curve represents the RMST for that group. p-values were calculated using a permutation test (1,000 iterations) for the difference in RMST between groups. Wild Type: n = 195; IHC-loss: n = 19.

Table S1. Clinicopathological Features According to Individual IHC Marker Loss Status

Variable	BAP1 loss (n=12)	p vs WT	PBRM1 loss (n=7)	p vs WT	Wild type (n = 195)
**Clinical Characteristics**					
Age (years), mean ± SD	63.1 ± 13.9	—	66.6 ± 10.3	0.049	58.4 ± 11.5
Male sex, n (%)	6 (50.0)	0.077	4 (57.1)	—	151 (77.4)
BMI (kg/m²), mean ± SD	26.2 ± 5.1	—	27.5 ± 3.8	—	27.3 ± 3.9
Preop. hemoglobin (g/dL), mean ± SD	13.4 ± 1.5	—	12.0 ± 2.2	0.019	14.0 ± 1.8
Follow-up (months), mean ± SD	44.9 ± 35.4	0.048	61.9 ± 56.8	—	75.2 ± 51.5
**Pathological Features**					
Tumor size (cm), mean ± SD	7.8 ± 5.8	—	7.4 ± 3.7	0.024	4.7 ± 3.0
High grade (G3–4), n (%)	6 (50.0)	0.003	2 (28.6)	—	25 (12.8)
Tumor necrosis, n (%)	6 (50.0)	0.018	1 (14.3)	—	36 (18.5)
Advanced stage (pT3–pT4), n (%)	6 (50.0)	0.010	3 (42.9)	—	32 (16.4)
Vascular invasion, n (%)	3 (25.0)	—	2 (28.6)	—	15 (7.7)
**Oncological Outcomes**					
Progression/recurrence, n (%)	5 (41.7)	—	4 (57.1)	0.032	38 (19.5)
Cancer-specific deaths, n (%)	3 (25.0)	—	1 (14.3)	—	20 (10.3)
Overall deaths, n (%)	3 (25.0)	—	1 (14.3)	—	36 (18.5)

p-values: Mann-Whitney U test (continuous) or Fisher exact test (categorical) vs. wild-type. All comparisons are exploratory. No multiple comparisons correction applied. BAP1: BRCA1-associated protein 1; PBRM1: polybromo-1; WT: wild-type.
Variables that did not reach statistical significance are indicated by "—".

Table S2. Exploratory Subgroup Analysis — Locally Advanced Disease (pT3–pT4)

The pT3–pT4 subgroup analysis (n = 40; IHC-loss n ≈ 9, wild-type n ≈ 31) is presented strictly as exploratory and hypothesis-generating. The multivariable Cox regression model for cancer-specific survival (CSS) was constrained to a single variable to avoid overfitting given only 4 CSS events in the IHC-loss arm — well below the minimum events-per-variable threshold (≥10 events per variable). The wide confidence interval (HR = 6.17, 95% CI 1.3–29.2, p = 0.022) reflects this statistical instability. Validation in larger prospective cohorts is required.

Variable	IHC-loss (n≈9)	Wild-type (n≈31)	p-value
Median time to progression (months)	7.0	46.0	—
Median CSS (months)	41.0	137.0	0.024
Median OS (months)	50.3	128.4	—

CSS: cancer-specific survival; OS: overall survival; HR: hazard ratio; CI: confidence interval. p-values from log-rank test (survival outcomes) or reduced multivariable Cox regression (HR). This analysis is strictly exploratory.
Variables that did not reach statistical significance are indicated by "—".

Table S3. Survival Outcomes and Multivariable Cox Regression Analysis in Patients with Advanced Disease (pT3–pT4).

Variable	HR (95% CI)	p value
IHC expression status (Ref. wild type)	6.17 (1.3–29.2)	0.022
Nuclear grade (1–2 vs 3–4)	—	—
Tumor necrosis (Ref. absence of necrosis)	—	—

Ref = reference category; HR = hazard ratio; CI = confidence interval.
Variables that did not reach statistical significance are indicated by "—".
